# Quasispecies evolution of the prototypical genotype 1 porcine reproductive and respiratory syndrome virus early during *in vivo* infection is rapid and tissue specific

**DOI:** 10.1007/s00705-017-3342-0

**Published:** 2017-03-30

**Authors:** Zen H. Lu, Xinglong Wang, Alison D. Wilson, Daniel L. W. Dorey-Robinson, Alan L. Archibald, Tahar Ait-Ali, Jean-Pierre Frossard

**Affiliations:** 1 PAPRSB Institute of Health Sciences, Universiti Brunei Darussalam, Bandar Seri Begawan, Brunei; 2 The Roslin Institute and Royal (Dick) School of Veterinary Studies, University of Edinburgh, Edinburgh, UK; 3 Northwest A&F University, Xianyang, China; 4 Animal and Plant Health Agency, Preston, UK

## Abstract

Porcine reproductive and respiratory syndrome virus (PRRSV) is a major infectious threat to the pig industry worldwide. Increasing evidence suggests that microevolution within a quasispecies population can give rise to high sequence heterogeneity in PRRSV; potentially impacting the pathogenicity of the virus. Here, we report on micro-evolutionary events taking place within the viral quasispecies population in lung and lymph node 3 days post infection (dpi) following experimental *in vivo* infection with the prototypical Lelystad PRRSV (LV). Sequence analysis revealed 16 high frequency single nucleotide variants (SNV) or differences from the reference LV genome which are assumed to be representative of the consensus inoculum genome. Additionally, 49 other low frequency SNVs were also found in the inoculum population. At 3 dpi, a total of 9 and 10 SNVs of varying frequencies could already be detected in the LV population infecting the lung and lymph nodes, respectively. Interestingly, of these, three and four novel SNVs emerged independently in the two respective tissues when compared to the inoculum. The remaining variants, though already present at lower frequencies in the inoculum, were positively selected and their frequency increased within the quasispecies population. Hence, we were able to determine directly from tissues infected with PRRSV the repertoire of genetic variants within the viral quasispecies population. Our data also suggest that microevolution of these variants is rapid and some may be tissue-specific.

**Electronic supplementary material:**

The online version of this article (doi:10.1007/s00705-017-3342-0) contains supplementary material, which is available to authorized users.

## Introduction

Porcine reproductive and respiratory syndrome virus (PRRSV) is a major infectious threat to the pig industry worldwide [33, 34]. The resulting disease (PRRS) is characterized by reproductive failure in sows and respiratory distress in young growing pigs [47, 54]. PRRSV, which taxonomically is a member of the family *Arteriviridae*, order *Nidovirales*, has been found to exist ubiquitously in almost all affected swine populations [41]. The virus has a positive-sense RNA genome of approximately 15 kb that encodes eight open reading frames which can be translated into at least 14 and 8 non-structural and structural proteins, respectively [27, 43]. PRRSV also has a specific cell tropism with a preference for cells of the monocyte/macrophage lineage, infecting subsets of differentiated macrophages in lungs (*i.e.* alveolar macrophages), lymphoid tissues and placenta [44]. A model of PRRSV entry and genome release has been reviewed elsewhere [45].

PRRSV is a rapidly evolving virus [13, 31]. The two genotypes, the European genotype 1 (prototype strain Lelystad) and the North American genotype 2 (prototype strain VR-2332) share about 60% nucleotide identity [2, 32]. However, considerable genetic variability exists also within both genotypes [3, 18, 53]. Furthermore, increasing evidence suggests that microevolution within a quasispecies population [19] can give rise to high sequence heterogeneity in PRRSV, potentially impacting the pathogenicity of the virus [5, 15, 23, 24]. Genetic and antigenic drift resulting from the dynamics of such mixed viral populations are likely to be of clinical relevance in terms of virulence and pathogenesis [9, 19].

Here, we assessed microevolutionary events taking place in a population of the prototypical Lelystad PRRSV in different tissue compartments of a single pig at 3 dpi.

## Materials and methods

### Experimental setup

Specific-pathogen-free seven week old piglets were infected with 10^5^ TCID_50_ PRRSV Lelystad strain (LV), as described by Morgan *et al* (2013) [30]. Briefly, the PRRSV LV strain was isolated from a clinical case of PRRS in the Netherlands in 1991 [47], and the inoculum used was from passage eight of this virus, performed in primary porcine alveolar macrophages. A portion of the inoculum was retained at -80°C. Serum, lung and mediastinal lymph node samples were collected at post-mortem, 3 dpi and stored in RNA*later* (Ambion).

RNA was extracted from the inoculum; tissues (lung and lymph node) and serum of 3 dpi pigs. Lung and lymph node total RNA were extracted using TRizol (Thermofisher) and the RNeasy kit (Qiagen) according to the manufacturer’s instructions. RNA from the inoculum and serum were extracted with the Viral Spin RNA kit (Qiagen) according to the manufacturer’s instructions. The quality and quantity of the extracted RNA was analyzed with the 2100 Bioanalyzer (Agilent Technologies). For RNA isolated from tissues, quantitative RT-PCR was performed, as previously described, to evaluate the PRRSV load. The selection of the 3 dpi pig from which lung and lymph node RNAs were processed for the RNA-seq was based on the following: i) the quality of RNA, as assessed with a RNA index number between 8 and 9 and ii) the Ct values of PRRSV RNA being between 20 and 28 [1] (Online Resource 1). For rRNA depletion, the Ribo-Zero kit (Illumina) was used. The paired-end RNA libraries were constructed and validated using the Truseq stranded Illumina library preparation kit (Illumina), as described previously [24]. Sequencing was performed on an Illumina HiSeq2500 and MiSeq platforms at Edinburgh Genomics (http://genomics.ed.ac.uk) and the Animal and Plant Health Agency (http://www.gov.uk/government/organisations/animal-and-plant-health-agency), respectively. All Illumina sequencing reads are available at EBI with the accession number PRJEB16756.

### Data analysis

For the downstream bioinformatics analysis, a strategy similar to that reported previously [24] was adopted. Briefly, the raw Illumina paired end data were first pre-processed to remove as much of the “contaminating” host (pig) RNA sequences as possible. These ‘pig-free’ sequence reads from the inoculum were then mapped to the reference LV genome (Genbank accession number M96262) using the software BWA (v0.7.5a) [20]. To reduce potential errors introduced by the excessive sequencing depth, only 10% of the total mapped reads with Phred-scaled alignment scores higher than 100 were randomly subsampled and retained for further processing whereby duplicates, secondary mappings and split reads, *i.e.* those comprising artifacts and/or the transcription-regulating sequence (TRS-) containing subgenomic RNA (sgRNAs) fragments, were removed. High quality variants (Q_phred_>20 and supported by at least three reads) including both single nucleotide variants (SNVs) and indels, if any, within the inoculum population were identified from the subsampled mapping using two separate programs; namely Samtools [21] and the low allele frequency variant caller, LoFreq [48]. Variants from both programs were compared using Bcftools [21] and differences inspected manually in the Integrative Genomics Viewer [38]. A final consensus sequence of the dominant PRRSV LV inoculum was constructed using high frequency variants (*i.e.* those with allele frequencies higher than 0.5). This consensus sequence of the inoculum next served as the reference PRRSV genome in the mapping and search for variants within not only the inoculum population but also in the PRRSV populations present in the lung and lymph nodes at 3 dpi. Both mapping and variant identification followed the same procedures as used in the analysis of the inoculum except that no down-sampling and removal of split reads were performed for the data derived from the tissue samples. The relatively low sequencing depth (Online Resource 2b) observed in the tissue-derived viral genomes required the omission of such processing to enable the capturing of as many of the variants as possible, especially the low frequency ones [37, 42].

### Variant validation

The nucleotide variants identified using next generation sequencing were validated using an alternative method involving targeted PCR amplification and Sanger sequencing. Briefly, the forward and reverse primer pair used to validate the variants was 5’-(2553)-TCCACAACGACCCTTGTGAG and 5’-(3112)-GCTTGGAGGCACTGTTCATATAC respectively. The bracketed positions of the primers are based on those of the reference LV genome. RNA isolated from lymph node, lung (supernatant of tissue homogenates) and serum at 3 dpi were reverse-transcribed into cDNA with the reverse primer and using the TaqMan reverse transcription system (Thermofisher) in a 20 µl reaction mixture. PCR amplification was conducted using FastStart Taq DNA Polymerase (Roche) with 1µl of cDNA. The reaction was run for 34 cycles at 95 °C denaturation for 45s, 58 °C annealing for 45s and 72 °C extension for 1-2 minutes. The amplified double-stranded DNA products were cloned into a pGEM-T vector (Promega) with T4 ligase and both the PCR and cloned products were sequenced using the ABI PRISM Big Dye terminator cycle sequencing ready reaction kit (Edinburgh Genomics, UK). Ten or more clones were analyzed until all sequence variants were identified and validated.

## Results

### Inoculum quasispecies population

Although the inoculum used in the present study was derived from the prototypical Lelystad PRRSV, we decided to not only re-sequence the virus but also to determine the exact constituents of the viral population. A depth well in excess of 95000x was achieved initially in the raw reads of the LV inoculum. However, only 10% of the high scoring mapped reads were randomly retained and further processed to yield a final sequence depth of approximately 840x (Online Resource 2a). Although 90% of the high quality mapped reads were not retained for subsequent analyses, 840x genome coverage should provide near complete representation of the full length genomic RNA of the PRRSV LV. However, for technical reasons the reads do not cover the first eight and last two nucleotides, at the 5’ and 3’ ends, respectively. No *de novo* assembly was attempted since the high quality mapping covered the “entire” genome with no gaps or indels found and the GC profile (averaging ~53%) of the viral genome was expected to pose no biases to the Illumina sequencing (Online Resource S2a). When compared to the reference LV genome, a total of 16 single nucleotide variants (SNVs), representative of the inoculum LV population, were identified (Table 1). Of these, five resulted in non-synonymous changes to the coding sequences. These 16 common variants were then used to construct the consensus genomic sequence of the inoculum LV strain.

**Table 1:** Dominant SNVs representative of the consensus inoculum LV strain when compared to the reference LV

Pos	Ref	Alt	Consequence	Residue Change	Gene	AF	Type
623	C	U	Synonymous	-	NSP1 (ORF1a)	0.69	Ti
2282	G	A	Synonymous	-	NSP2 (ORF1a)	0.99	Ti
2714	A	G	Synonymous	-	NSP2 (ORF1a)	0.99	Ti
3063	G	A	Non-Synonymous	D948N	NSP2 (ORF1a)	0.93	Ti
3971	U	C	Synonymous	-	NSP2 (ORF1a)	0.96	Ti
4535	U	C	Synonymous	-	NSP3 (ORF1a)	0.99	Ti
4584	U	C	Non-Synonymous	S1455P	NSP3 (ORF1a)	0.80	Ti
6188	A	C	Synonymous	-	NSP5 (ORF1a)	0.99	Tv
6875	G	A	Synonymous	-	NSP7 (ORF1a)	0.99	Ti
8383	C	U	Synonymous	-	NSP9 (ORF1b)	0.67	Ti
8956	A	C	Synonymous	-	NSP9 (ORF1b)	0.99	Tv
9682	G	A	Synonymous	-	NSP10 (ORF1b)	0.99	Ti
10624	U	C	Synonymous	-	NSP10 (ORF1b)	0.85	Ti
11880	C	U	Non-Synonymous, Non-Synonymous	P29S, A27V	GP2a, GP2b (ORF2)	0.99	Ti
13602	G	A	Non-Synonymous, Non-Synonymous	D37S, R35Q	GP5, GP5a (ORF5)	0.98	Ti
13603	A	G	Non-Synonymous	D37S, R35Q	GP5, GP5a (ORF5)	0.99	Ti

Pos: position on the reference genome, Ref: reference nucleotide, Alt: SNV, AF: allele frequency; Ti: transition mutation; Tv: transversion mutation

In addition, another 49 low frequency SNVs with intra-strain population frequencies ranging from approximately 0.006 to 0.3 (Online Resource 3) were also detectable within the heterogeneous quasispecies population when the sequencing reads were mapped back against the newly constructed consensus genome of the inoculum. Although both the common and low frequency SNVs were distributed throughout different regions of the genome, a high number of them (13 out of the 49 SNVs) and many of which were non-synonymous changes, were found to concentrate in regions of the *nsp2* gene where known, or predicted, B-/T-cell epitopes are located [24].

### Viral micro-evolutionary dynamics *in vivo*

**Fig. 1:**
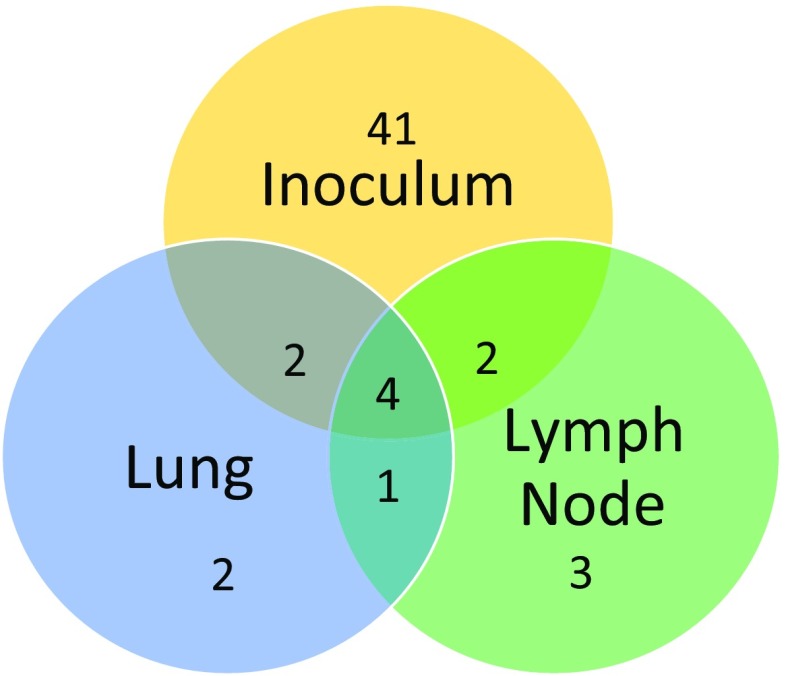
Venn diagram representing SNVs within the PRRSV population identified in the inoculum prior to infection and in tissues (lung and lymph node) at 3 dpi

In order to gain insights into the micro-evolutionary dynamics of the viral quasispecies, we searched for variants present within the entire viral population isolated from lung and lymph node of a single piglet experimentally infected with the LV inoculum. The criteria for selecting the piglet are described in the materials and methods section. The comparisons between tissues were particularly significant since they were from the same animal, showing evolution of the virus from just one inoculum, in the same macro-environment. At 3 dpi, the total viral yields from the two tissues differed by more than three-fold as indicated by the ~15x and ~50x average sequence coverage respectively (Online Resource 2b). While many experimental factors could have contributed to such a difference, tissue tropism of the PRRSV quasispecies population could be at play here. A total of 9 and 10 SNVs were found in the PRRSV population residing in the two tissues, respectively (Table 2 & Online Resource 4). Interestingly, six of these SNVs were newly emerged variants that we had not detected in the very high coverage sequence data from the inoculum population; with two found only in PRRSV isolated from the lung, three found only in the lymph node associated PRRSV and one shared by both (Fig. 1 & Online Resource 4). The remaining eight SNVs found in both tissues were already present in the quasispecies population of the inoculum. However, the frequencies of these eight SNVs had increased rapidly from ~0.01-0.3 in the inoculum to ~0.3-0.8 in the two tissues. The increased frequency of these SNVs could be the result of positive selection for nucleotides that could benefit the virus during its earlier infection cycles.

**Table 2:** LoFreq detected SNVs present in the LV population isolated from tissues 3 dpi

Pos	Ref	Alt	Consequence	Residue Change	Gene	AF (tissue)	Type
215	U	G	Synonymous	-	TRS	0.11 (LN)	Tv
623	U	C	Synonymous	-	NSP1 (ORF1a)	0.72 (L)	Ti
1068	G	A	Non-Synonymous	G283S	NSP1 (ORF1a)	0.38 (L)	Ti
2220	A	G	Non-Synonymous	S668G	NSP2 (ORF1a)	0.31 (L)	Ti
2999	U	C	Synonymous	-	NSP2 (ORF1a)	0.6 (L)	Ti
3063	A	G	Non-Synonymous	D948N	NSP2 (ORF1a)	0.4 (L) 0.3 (LN)	Ti
3971	C	U	Synonymous	-	NSP2 (ORF1a)	0.72 (L) 0.24 (LN)	Ti
4584	C	U	Non-Synonymous	S1455P	NSP3 (ORF1a)	0.8 (L) 0.4 (LN)	Ti
5918	C	U	Synonymous	-	NSP5 (ORF1a)	0.8 (L) 0.39 (LN)	Ti
5954	C	U	Synonymous	-	NSP5 (ORF1a)	0.23 (LN)	Ti
6674	C	U	Synonymous	-	NSP7 (ORF1a)	0.19 (L) 0.47 (LN)	Ti
8383	U	C	Synonymous	-	NSP9 (ORF1b)	0.31 (LN)	Ti
9190	U	C	Synonymous	-	NSP9 (ORF1b)	0.41 (LN)	Ti
10247	A	U	Non-Synonymous	T952S	NSP10 (ORF1b)	0.37 (LN)	Tv

Pos: position on the inoculum LV genome, Ref: nucleotide in the inoculum, Alt: low frequency SNV, AF: allele frequency, L: lung, LN: Lymph node; Ti: transition mutation; Tv: transversion mutation

Interestingly, approximately 1% (Online Resource 3) of the inoculum quasispecies population may actually harbor a SNV (nucleotide 217) on the leader segment of the hexanucleotide TRS. However this SNV was no longer detectable *in vivo*. Instead, a second such SNV at nucleotide 215 emerged in the PRRSV population isolated from lymph nodes at 3 dpi. The potential effect of SNVs on this regulatory element should not be overlooked even though the TRS of PRRSV sgRNAs are known to lack absolute conservation [26, 51].

Although the modest sequence coverage (>30X) of some regions of the virus in the two tissues might have prevented the detection of low frequency variants (especially those supported by less than three reads), when compared together, the SNV composition within the PRRSV quasispecies population in the inoculum, lungs and lymph nodes clearly shows that these microevolutionary events are rapid. The mutation rate of PRRSV [11–13] and other single-stranded RNA viruses [10], based on the studies of a single ORF, has previously been estimated to range from 1.8 x 10^−3^ to 5.8 x 10^−3^ substitution/site/year.

The appearance of different variants in the two different tissues may reflect the timing of the initial mutation events or some tissue-specificity in terms of which variants arise or proliferate. For variants present in the initial inoculum population but with subsequent differences in frequency (including presence/absence) between the two tissues, the differences may arise from which variants reached the relevant tissue from the inoculation site or again some tissue specificity in terms of survival. Furthermore, the nucleotide changes in the majority of the SNVs detected here were of the transition substitution type; providing perhaps a hint of the directionality of the nucleotide-specific mutation in the different tissues.

**Fig. 2:**
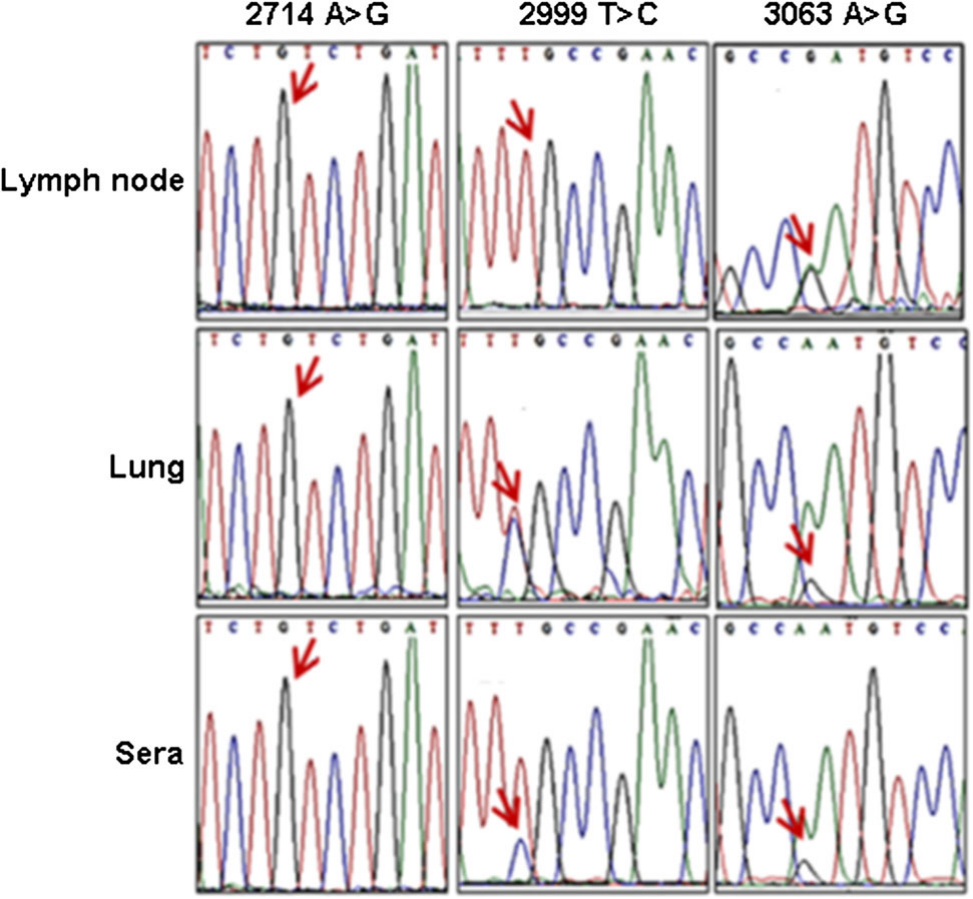
Validation of 3 SNVs using Sanger sequencing. RNA from lung, lymph node and serum were isolated, reverse-transcribed and analyzed by PCR. Traces were selected around SNV positions 2714, 2999 and 3063 (red arrows)

Validation of 3 selected SNVs at nucleotide position 2714, 2999 and 3063 (Table 3) was performed using PCR and Sanger sequencing in lung, lymph node and the serum (Fig. 2). The use of the serum for SNV validation allowed us to assess whether a PRRSV variant was circulating in the bloodstream or fixed in a tissue. Whereas most of the SNVs were consistently detected in the serum, lung and lymph node, SNV 2999 appeared fixed in the lymph node only. Furthermore, SNV 2714 did not undergo significant variation in the tissues tested. These data suggest that the SNVs obtained using Sanger sequencing were in agreement, at least qualitatively, with the data generated using next generation sequencing.

**Table 3:** Allelic frequencies of SNVs at nucleotide position 2714, 2999 and 3063, gained using next generation data analysis

SNVs	2714 (A>G)	2999 (T>C)	3063 (A>G)
Inoculum	0.995	1	0.931
Lymph node	1	1	0.3
Lung	1	0.6	0.4
Comments	Not altered	Specifically altered in lung	Altered in all tissues

## Discussion

Several studies have looked at quasispecies formation following PRRSV infection, *in vitro* and *in vivo* [5, 6, 15, 39, 40, 52]. However, they have focused on specific genetic regions – usually ORF5 – and to date no study has looked at differences *in vivo* across the whole genome. NGS provides a rapid way to sequence viral genomes [23]. Here, we generated the full genome sequence of PRRSV LV directly from tissues of an infected pig. The massive sequencing depth and coverage of the genome provided by NGS allowed not only a systematic analysis of PRRSV genome variation *in vivo* but also confident identification of low frequency variants present in the PRRSV quasispecies populations. However, due to the limitation of the current methodologies in distinguishing precisely between SNVs arising from either genomic or sgRNA, it is important to note that this study is in fact representative of the entire variants present in the quasispecies population.

The differences in the variants and variant frequencies identified between the PRRSV populations present in lung and lymph node may be indicative of some tissue specificity or selection. This has not previously been examined in other studies. Alternatively, the differences may reflect the timing of mutation events and/or which viral variants reached the tissues from the inoculation site. In the lung, alveolar macrophages are the primary target cells for PRRSV, but pneumocyte type II cells can also be infected [16]. It is suggested that PRRSV enters the mediastinal lymph nodes from the apical and medial lung lobes via draining lymphatic vessels [14]. Different types of macrophages, as well as dendritic cells may be infected in the lymph node [7]. As infection spreads to a new tissue, it is likely that a small number of virions are initially involved, and that any mutations in that population are therefore reflected later in the quasispecies. Some of the mutations may provide increased fitness for infection, replication, or propagation in the new cell type, thereby becoming established in the quasispecies as manifested by the increasing dominance of some SNVs in the current work.

The PRRSV genes in which the new SNVs were found all include some element of interaction with the host, either directly or through the immune response. NSP1 alters IFN-β expression [36]; 285 host cellular proteins have been identified that interact with NSP2 in PRRSV-infected cells [46]; NSP5 interacts with the host membrane, anchoring the viral RNA-synthesizing machinery to modified intracellular membranes [36], and it features T-cell epitopes [28]; NSP7 features antigenic epitopes important in induction of host humoral immune response [4]; NSP9 and NSP10 contain potential T-cell epitope [35]. It is therefore possible that one or more of the SNVs identified is linked to a change favorable for infection of, or replication in, different cell types.

It is postulated that quasispecies evolution in an infected animal is also affected by the host immune responses, aiding in viral escape of the immune factors [50]. In this case, no significant antibody response is yet present at just 3 dpi, so any immune pressure on the virus population is due only to the innate cellular response. While it may previously have been assumed that the majority of changes in response to immune pressure would be found in structural genes, it is clear that some non-structural proteins generate antibody responses [36], and T-cell epitopes are found in non-structural genes as well [29]. It is therefore important to examine the whole genome of PRRSV when evaluating possible evolution in the face of immune responses in the pig.

In addition to antigenic and structural changes that may be induced by the amino acid changes, some synonymous nucleotide changes may also have an impact. In particular, nucleotide changes in the 5’ UTR may affect genomic replication, subgenomic RNA transcription, and mRNA translation [17]. Indeed, although the exact significance of nucleotide changes on regions such as the TRS is yet to be investigated, our preliminary data has shown that the nucleotide composition of the TRS may have an impact on the transcriptional rate of sgRNAs and hence affect the infectivity of the virus (data unpublished). Furthermore, some silent nucleotide changes within coding regions of the viral genome may also affect translational efficiency due to the different preferences of codon usage by the host cells [22], or through differences in RNA folding and structure [8, 49].

While other studies have identified heterogeneity in structural genes of PRRSV, for example in serum at 11 and 21 dpi [5], this is the first study to report heterogeneity in PRRSV as soon as 3 dpi in different tissues of an infected pig. Although some changes in structural genes were identified in the inoculum when compared to the published LV sequence, all of the variants identified in the pig tissues early after infection were located in non-structural genes. The concentration of variants in this region is unlikely to be the result of sequencing biases. As illustrated by the sequence read mapping (Online Resource 2b), the sequencing depth is generally even greater for the structural genes. Furthermore, the unevenly distributed sequencing depth cannot be attributed to any regions with unusually high or low GC content (Online Resource 2a). In fact, the viral RNA secondary structure has been suspected to be the cause [25].

Molecular clock estimates for PRRSV have predicted a rate of between 1.8 and 5.8 x 10^−3^ substitution/site/year [11–13]. However, the fact that multiple SNVs have emerged within the PRRSV quasispeicies population in three days of infection points to perhaps a faster rate of evolution. This difference may be due to the previous analyses being based solely on ORF3 or ORF7 sequences rather than the whole genome. As shown here, the majority of substitutions were found in other parts of the genome.

The NGS method provides a rapid way to sequence viral genomes even without prior knowledge of any of the viral genomic information. Full genomes were obtained for PRRSV directly from tissues of an infected pig. The high sequencing depth and coverage of the genome provided by NGS have not only allowed the analysis of genome variation *in vivo* but also confident identification of low frequency variants present in the quasispecies population. Further study of samples from *in vivo* PRRSV infections is therefore possible to elucidate the dynamic interplay between the rapidly evolving quasispecies and the host response to the viral infection.

## Electronic supplementary material

Below is the link to the electronic supplementary material.

Supplementary material 1 (PDF 301 kb)

Supplementary material 2 (PDF 547 kb)

Supplementary material 3 (XLSX 12 kb)

Supplementary material 4 (PDF 285 kb)
