# Supplementary material for: Quasispecies evolution of the prototypical genotype 1 porcine reproductive and respiratory syndrome virus early during in vivo infection is rapid and tissue specific
Source: Arch Virol. 2017 Mar 30;162(8):2203–10. doi: 10.1007/s00705-017-3342-0 (PMC5506507; doi:10.1007/s00705-017-3342-0)
Supplement: Supplementary file 1 — Supplementary material 1 (PDF 301 kb) [file 705_2017_3342_MOESM1_ESM.pdf]

(a)

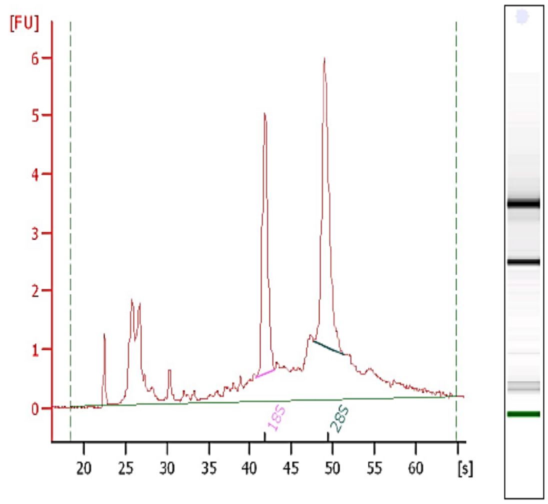

(b)

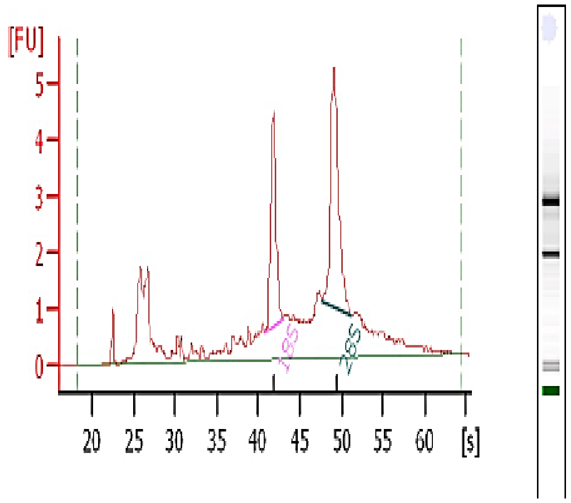

Figure S1: 2100 Bioanalyzer (Agilent Technologies) electrophoregramme: (a) Lymph node RNA with RIN of 8.5 and (b) Lung RNA with RIN of 8.0.
