# Supplementary material for: Quasispecies evolution of the prototypical genotype 1 porcine reproductive and respiratory syndrome virus early during in vivo infection is rapid and tissue specific
Source: Arch Virol. 2017 Mar 30;162(8):2203–10. doi: 10.1007/s00705-017-3342-0 (PMC5506507; doi:10.1007/s00705-017-3342-0)
Supplement: Supplementary file 2 — Supplementary material 2 (PDF 547 kb) [file 705_2017_3342_MOESM2_ESM.pdf]

(a)

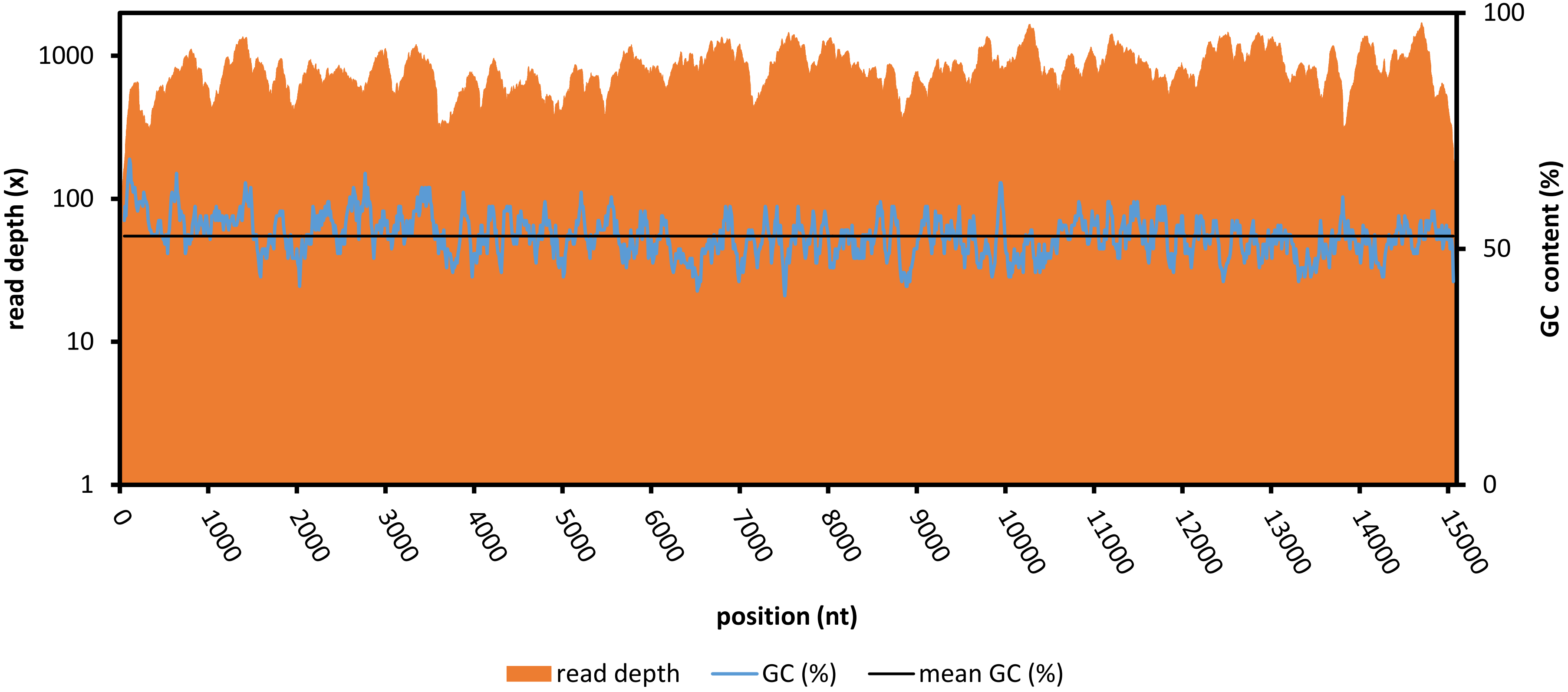
read depth (x)
GC content (%)
position (nt)
1000
100
10
1
100
50
0
0
1000
2000
3000
4000
5000
6000
7000
8000
9000
10000
11000
12000
13000
14000
15000
read depth
GC (%)
mean GC (%)

(b)

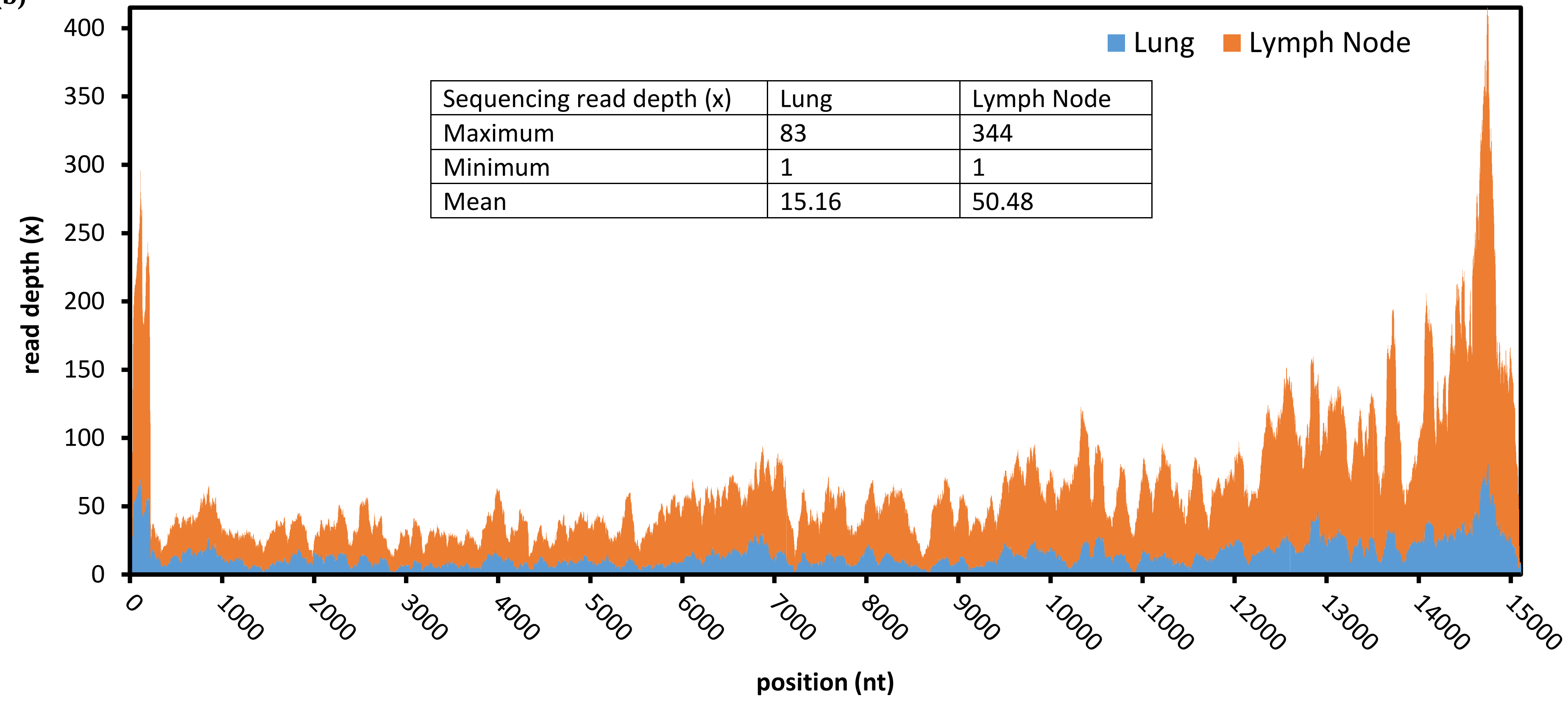

Figure S2: NGS of PRRSV LV inoculum prior and 3 days post infection (dpi). (a) Mapping of the inoculum sequencing reads to the reference LV. Coverage plot depicts only reads representing mainly the genomic RNA. The GC content (blue line) (based on 100 nt sliding windows) was also plotted to ensure the absence of biases across the viral genome. (b) Mapping of the PRRSV's total RNA sequencing reads extracted from lung and lymph node 3 dpi to the reference inoculum. The peak at the 5'UTR and the increasing coverage towards the 3'end were the result of the accumulation of nested sgRNAs, Various calculations for the sequencing depth of the viral genome isolated from the two tissues were also tabulated.
