# Supplementary material for: Quasispecies evolution of the prototypical genotype 1 porcine reproductive and respiratory syndrome virus early during in vivo infection is rapid and tissue specific
Source: Arch Virol. 2017 Mar 30;162(8):2203–10. doi: 10.1007/s00705-017-3342-0 (PMC5506507; doi:10.1007/s00705-017-3342-0)
Supplement: Supplementary file 4 — Supplementary material 4 (PDF 285 kb) [file 705_2017_3342_MOESM4_ESM.pdf]

(a)

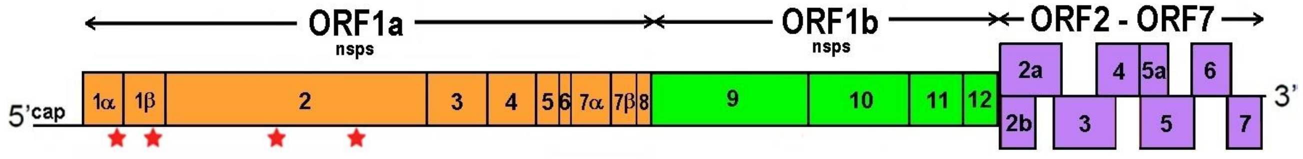

(b)

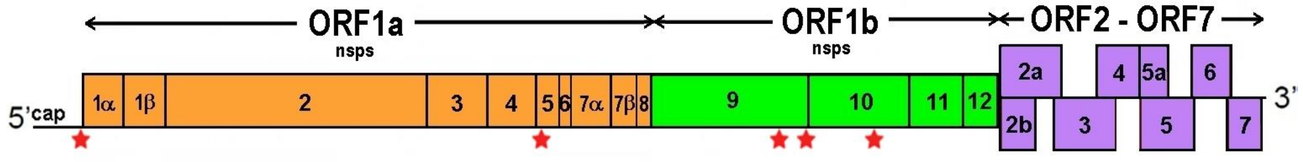

(c)

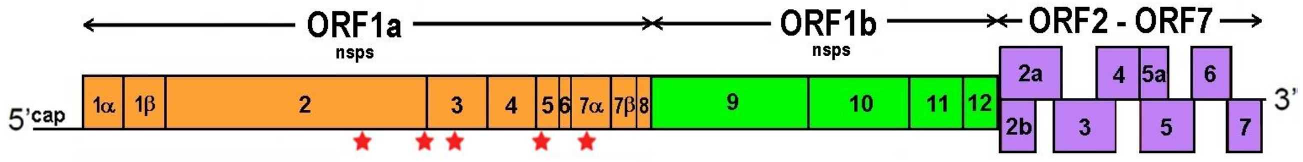

Figure S3: Single nucleotide variants (SNVs) detected in the quasispecies population of PRRSV LV isolated from the pig tissue at 3 days post infection (dpi). The stars illustrate the relative positions of the SNVs identified in (a) lung versus inoculum, (b) lymph node versus inoculum, and (c) both tissues versus inoculum.
